# Expectation Modulates Hedonic Experiences and Midbrain Responses to Sweet Flavor

**DOI:** 10.1523/JNEUROSCI.1121-25.2026

**Published:** 2026-03-02

**Authors:** Elena Mainetto, Margaret L. Westwater, Hisham Ziauddeen, Kelly M. J. Diederen, Paul C. Fletcher

**Affiliations:** ^1^Department of Psychiatry, University of Cambridge, Cambridge CB2 0SZ, United Kingdom; ^2^Donders Institute for Brain, Cognition and Behaviour, Radboud University, Nijmegen 6525 HT, The Netherlands; ^3^Department of Psychiatry, University of Oxford, Warneford Hospital, Oxford OX3 7JX, United Kingdom; ^4^Mental Health Service, Fiona Stanley Hospital, Perth, Western Australia 6150, Australia; ^5^Department of Psychosis Studies, Institute of Psychiatry, Psychology and Neuroscience, King’s College London, London SE5 8AF, United Kingdom; ^6^Wellcome Trust MRC Institute of Metabolic Science, University of Cambridge, Cambridge CB2 0QQ, United Kingdom; ^7^Cambridge and Peterborough NHS Foundation Trust, Cambridge CB21 5EF, United Kingdom

**Keywords:** dopamingergic, expectancies, functional MRI, gustatory reward, sugar

## Abstract

Non-nutritive sweeteners are sugar substitutes that may promote weight management by reducing an individual's calorie intake. It is, however, unclear whether (1) sugar and non-nutritive sweetener elicit distinct orosensory responses in the human brain and (2) whether the neural responses to these flavors are modulated by expectancy. Addressing these questions has direct relevance to our understanding of food choice behavior and how it may be modified in dietary interventions. We screened *N* = 99 healthy adults of either sex to select a sample (*N* = 27; *M*[SD]_age _= 24.25[2.94] years) who reported similar perceptual experiences of sugar and sweetener, thus removing a potential confound of sensory differences, for fMRI scanning. While scanning, they received sugar- and artificially sweetened beverages in two conditioning paradigms, which manipulated participants’ expectation of flavor delivery: first in a probabilistic and second in a deterministic way. Participants’ ability to accurately distinguish sugar from non-nutritive sweetener depended largely on their expectations, which also significantly affected the perceived pleasantness of each flavor. Expectation altered brain responses to flavor delivery during the deterministic task only, where the (mistaken) expectation of sugar significantly increased midbrain responses to sweetener compared with when sweetener was expected. Trial-wise confidence and pleasantness ratings differentially scaled with brain responses to sugar and sweetener delivery. These results highlight the importance of expectancy in both the behavioral and neural encoding of sweet flavor, particularly when sensory information is unreliable. The expectation of sugar appears to increase the subjective value of noncaloric sweetener, which may result from flavor-nutrient conditioning that preferentially reinforcers sugar.

## Significance Statement

Artificial sweeteners have become common alternatives to sugar-sweetened beverages. The perceived reward from sweet flavor depends not only on the sweetener but also on our expectations of its resulting pleasantness. However, it remains unknown if shared brain circuits encode sugar and non-nutritive sweetener—and our expectations surrounding them. Here, we examined brain responses to sugar- and artificially sweetened beverages in healthy humans who could not reliably discriminate them, and we manipulated their expectation of flavor delivery. Expectation altered participants’ accuracy and perceived pleasantness of each flavor, where the expectation of sugar increased midbrain responses and perceived pleasantness of artificial sweetener. The rewarding effects of sugar appeared to exceed those of sweetener, which may reflect flavor-nutrient conditioning that shapes food choice behavior.

## Introduction

Global trends in overweight and obesity have catalyzed research into the reinforcing properties of highly palatable foods, and sugar remains a common target of policy and intervention efforts surrounding weight management (Hsiao and Wang, 2013). Some efforts promote non-nutritive sweeteners, such as saccharin, sucralose, or aspartame (henceforth “sweeteners”), as sugar substitutes that reduce excessive caloric intake but maintain a similarly rewarding flavor. However, little is known about how the human brain encodes sugar and sweetener consumption.

Preclinical neuroscience findings indicate distinct neural pathways that signal the hedonic and nutritive value of sugar, which broadly correspond to the ingestive and postingestive effects of consumption. The hedonic value of sweet flavor arises from orosensory inputs that depolarize taste receptors, which innervate the nucleus tractus solitarius and subsequently to the thalamus and primary gustatory cortex (Ogawa, 1994). Insula subregions then target reward-related brain areas, including the ventromedial prefrontal cortex and ventral striatum (Haber, 2011). Indeed, both sweetener and sugar ingestion augments nucleus accumbens dopamine release, even in sham-fed animals (Hajnal et al., 2004). In contrast, the nutritive value of sugar is signaled by melanin-concentrating hormone–expressing neurons in the lateral hypothalamus that target the dorsal striatum (Domingos et al., 2013), which selectively responds to intragastric sugar (Ren et al., 2010; Tellez et al., 2016). This central pathway also receives peripheral input from hepatic portal vein sensors via the gut–brain axis (Zhang et al., 2018).

While rodents are typically exposed to both sweetener and sugar, few studies have investigated neural responses to both flavors in humans. Meta-analytic findings indicate robust anterior insula, frontal operculum, and dorsal striatal responses to sugar delivery, yet caloric sweet taste did not reliably elicit ventral striatal responses (Roberts et al., 2020). Findings suggest similar gustatory cortex, thalamic, and precuneus responses to tasting sucrose and sweetener within-subjects, yet sucrose delivery augmented dorsal striatal, cingulate, and prefrontal cortex activation relative to sweetener (Frank et al., 2008; Haase et al., 2009; Green and Murphy, 2012). Ventral striatal responses to sugar delivery may be modulated by sensory-specific satiety, whereas greater amygdala activation following sweetener versus sugar delivery is reportedly independent of satiety (Smeets et al., 2011).

Another challenge for human studies of sweeteners relates to potential differences in their expected value relative to sugar. Sweeteners have an intense sweetness and different affinities for “sweet” taste receptors (Li et al., 2002) that may elicit qualitatively different perceptions than sugar. Moreover, sweeteners also bind to “bitter” T2 taste receptors, often making them easily distinguishable from sugar and less pleasant. These orosensory properties may lead one to anticipate sweeteners to be less palatable, thereby reducing their reward value through reinforcement learning (Diederen and Fletcher, 2021). Midbrain dopaminergic prediction error (PE) signals, which code the difference between expected and actual value, underlie reinforcement learning, where both the sign and magnitude of a PE contribute to the reward value of a given outcome. Therefore, the apparently less rewarding effects of sweeteners may relate to differences in not only their associated PEs and their intrinsic hedonic properties but also learning-based expectation.

Here, we characterized neural responses to sugar- and sweetener-sweetened beverages while accounting for potential differences in perceptual experiences and examining the influences of reward expectancies. Critically, we recruited participants who were unable to distinguish these flavors above chance level, thus ensuring that expectancies could be manipulated independently from perceptual experience. Trial-specific cueing engendered varyingly strong expectations about whether the ensuing liquid delivery would contain sugar or sweetener under probabilistic and deterministic conditions. We predicted that expectation would alter behavioral performance and neural responses to sugar and sweetener across both gustatory and reward networks. We expected similar neural responses to each flavor across gustatory regions, aligning with participants’ poor discriminability; however, the expectation of sugar would increase reward network activation and pleasantness, whereas sweetener expectation would attenuate them, irrespective of the flavor delivered. Exploratory analyses examined parametric effects of confidence and pleasantness ratings during the probabilistic and deterministic tasks, respectively.

## Materials and Methods

### Participants

Ninety-nine (*n* = 54 female; *M*[SD]_age_ = 23.75[3.66] years) participants were recruited from posted advertisements, university mailing lists, and social media in Cambridge. Eligible volunteers were healthy adults between 18 and 40 years of age with a body mass index (BMI) between 18.5 and 24.9 kg/m^2^. Exclusion criteria included the presence of a significant medical or psychiatric history, contraindications to MRI scanning (e.g., pregnancy, some metallic implants), being left-handed, current dieting, being a competitive sportsperson (>5 h high-intensity exercise per week), or having English language or communication difficulties that would impede understanding of the study procedures. Participants who disliked lemonade or did not consume sugar-sweetened beverages were also ineligible.

### Study procedures

Potential volunteers attended a 30 min screening session, which involved self-report questionnaires and a gustatory task (described below) that assessed participants’ ability to discriminate between the sugar and sweetener version of the same beverage, Sainsbury's Classic Lemonade and Sainsbury's Diet Lemonade. Participants refrained from eating or drinking anything except water for 1 h prior to the screening, and they were told that the visit would involve tasting the liquids that would be delivered in the subsequent MRI visit. The two lemonade products had the same composition apart from the sweetening agent. Critically, the classic lemonade did not contain non-nutritive sweetener in addition to sugar. As we sought to examine the effect of expectancies on gustatory perception, we limited our sample to participants who were relatively unable to discriminate between the two liquids. Individuals with a discrimination accuracy below 50% or above 60% were excluded, as accuracy values in these ranges would reflect poor task engagement or high discrimination between the two liquids, respectively. Although these exclusion criteria were disclosed to ineligible volunteers, eligible participants were not informed of the reason for their selection. Of the screening sample, 27 participants (*n* = 19 female; *M*[SD]_age_ = 24.25[2.94] years) were invited to the scanning session. Due to a lack of available effect size estimates, an a priori power analysis was not completed; however, the target sample size reflected recommendations for fMRI study sample sizes at the time of data collection (Poldrack et al., 2017; Szucs and Ioannidis, 2017).

MRI scan sessions began at ∼9:00 h, lasting 90 min. Participants were asked to refrain from eating or drinking anything (except water) 8 h prior to the study session, and they provided internal state ratings (hunger, thirst) and liking ratings for each liquid using a visual analog scale (VAS; 0, “Not at all”; 100, “Extremely”) at the start, midway point, and end of scanning. During each scan, participants completed probabilistic and deterministic conditioning tasks, which lasted 70 min in total. In each task, SU and SW liquids were paired with one of the two colors (green or purple). The color association was randomized and counterbalanced across participants. Participants were informed that the experiment included both short and long rest periods, and they were instructed to look at a fixation cross. After the MRI scan, participants completed validated self-report measures of depressive symptoms (Patient Health Questionnaire-9, PHQ-9; Kroenke et al., 2001), nonspecific psychological distress (Kessler Psychological Distress Scale K10; Kessler et al., 2003), physical activity (Besson et al., 2010), and eating behavior (Three-Factor Eating Questionnaire-Revised; Karlsson et al., 2000). The PHQ-9 indexes the frequency of depressive symptomatology over the previous 2 weeks using a four-point Likert scale (0, “Not at all”; 3, “Nearly every day”), with higher scores corresponding to greater severity (ranging from 0 to 27). Similarly, the K10 queries cross-cutting psychiatric symptoms over the previous 30 d on a five-point Likert scale (1, “None of the time”; 5, “All of the time”). Total scores may range from 10 to 50, where scores below 20 would indicate the likely absence of mental illness. The Recent Physical Activity Questionnaire measures daily physical activity over the past 4 weeks across three domains: at home, at work, and during leisure time. Responses are then used to estimate daily total energy expenditure and total time spent in any activity or sleep (hours/day). Finally, the Three-Factor Eating Questionnaire indexes three aspects of eating behavior—cognitive restraint, uncontrolled eating, and emotional eating—one a four-point Likert scale (4, “Definitely true”; 1, “Definitely false”).

All the participants provided signed, informed consent at the beginning of the screening session, and they were compensated £60 for their time. Participants who only completed the screening visits received £10. The study was approved by the ethics committee of the University of Cambridge (Ref. PRE.2017.058).

### Gustometer system

This study was conducted using a custom-built liquid delivery system created for use in MRI (Ziauddeen et al., 2016). Liquid volumes of 0.9 ml were delivered over 3 s via programmable syringe pumps. The pumps were controlled by task scripts, which were written in MATLAB (2016a; MathWorks). Each pump held one of the three 50 cc syringes, containing either sugar-sweetened lemonade, non-nutritive sweetened lemonade, or tap water, which was connected to a 10 m length of PVC plastic tubing (Thermo Fisher Scientific; 3 mm diameter, 0.75 mm wall thickness) that were held together in a sleeve. The tubes were affixed to a pacifier, which held them in the participant's mouth. The plastic tubing and syringes were all commercially manufactured products designed for clinical use. A new set of syringes and tubing were used for each participant and were discarded after single use for hygiene purposes.

### Screening—discrimination task

During the screening session, participants completed a two forced-choice task, in which they received two liquids from the pump system and rated whether the two flavors were the same or different. On each trial, the word “TASTE” appeared for 3 s on the screen to indicate the delivery of either sweetener or sugar. Participants were instructed to hold the liquid in their mouth until the word “SWALLOW” appeared on screen, which was also presented for 3 s. Then, they indicated whether they thought the two tastes were different or the same. Finally, participants rated how confident they were in their decision on a VAS scale (0, “Not at all”; 100, “Extremely”). They had 5 s to respond to each question using a mouse. This task consisted of 40 trials, separated by a 500 ms intertrial interval: 10 for congruous sugar–sugar, 10 for congruous sweetener–sweetener, 10 for incongruous sugar–sweetener, and 10 for incongruous sweetener–sugar. After every 10 trials, participants received a 1 ml rinse of water.

### MRI acquisition

MRI data were collected on a 3 T Siemens Prisma scanner that was fitted with a 32-channel head coil at the Cognition and Brain Sciences Unit in Cambridge. The following parameters were used for echo-planar imaging: repetition time (TR), 1,503 ms; echo time (TE), 32.40 ms; 2 mm isotropic voxels; slice thickness, 2 mm; flip angle 74°; field of view (FOV), 256 mm; matrix, 96 × 96; and 60 axial slices. We acquired 1,660 multiband echo planar T_2_*-weighted volumes (GE-EPI) with a multiband acceleration factor of 3 for the probabilistic task. For the deterministic task, 779 volumes were collected using the same multiband parameters. To improve localization of the functional data, a high-resolution T_1_–weighted anatomical scan was acquired during the same scan session (TR, 2,250 ms; TE, 3.02 ms; 1 mm isotropic voxels; slice thickness, 1 mm; flip angle, 9°; FOV, 192 mm; 192 slices).

### Neuroimaging—probabilistic task

**Figure 1.**
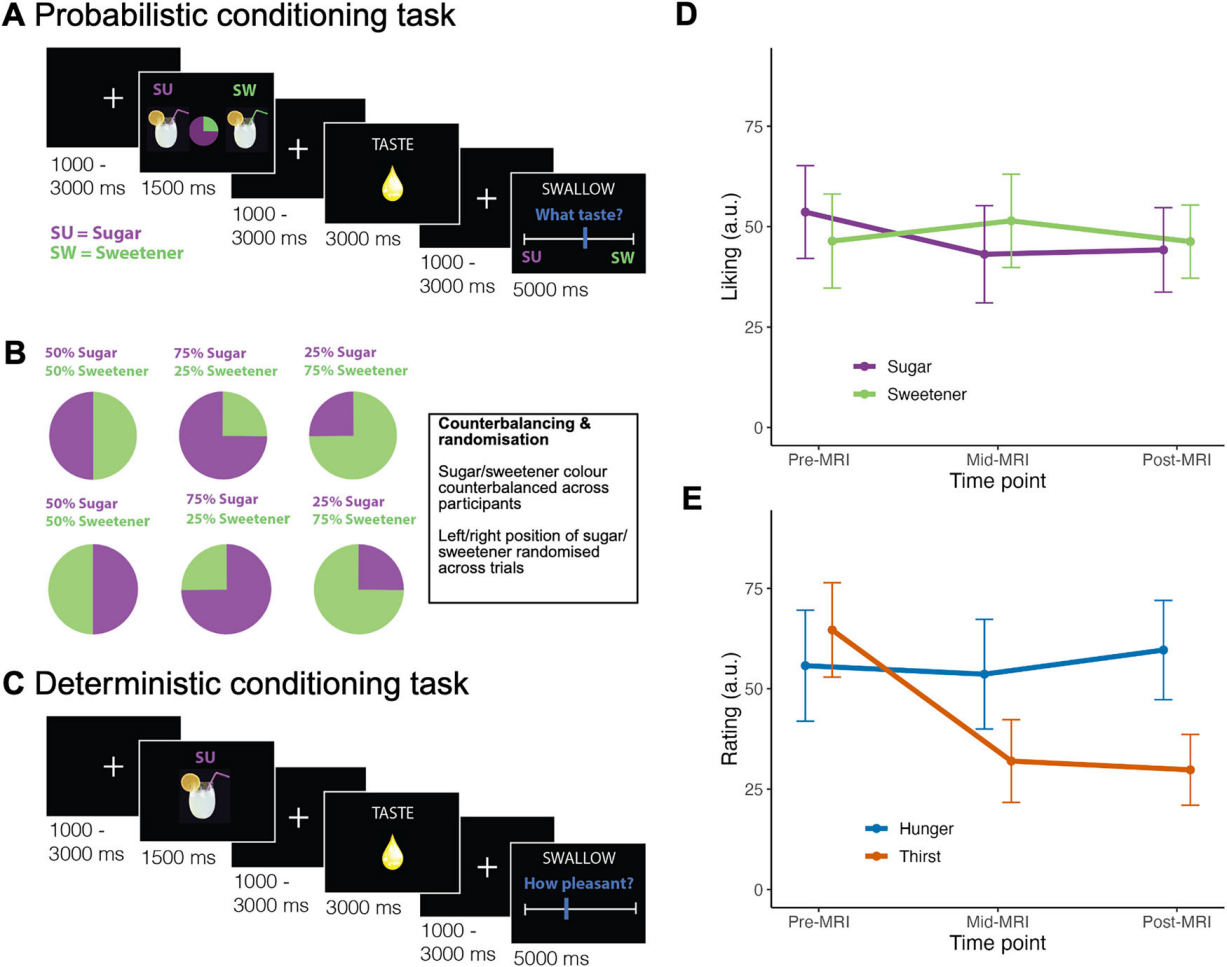
Probabilistic and deterministic conditioning task trial structure and internal state ratings. ***A***, Probabilistic conditioning task trial structure. A cue indicating the probability of sugar-sweetened lemonade and non-nutritive sweetener lemonade was presented prior to flavor stimulus delivery. Participants were instructed to hold the liquid in their mouth until the “SWALLOW” cue was presented, at which time they rated which flavor they received. ***B***, The associated color (green or purple) for each flavor was counterbalanced across participants, and the position of each flavor (left or right) on the slider was randomized across trials. ***C***, Deterministic conditioning task trial structure. On each trial, a cue depicting either sugar-sweetened lemonade or non-nutritive sweetener lemonade was followed by delivery of the cued liquid. However, cues were covertly violated on 50% of trials (except for the neutral water rinse). Participants were then instructed to swallow the liquid and rate the pleasantness of the flavor. ***D***, Liking ratings did not differ significantly between sugar and non-nutritive sweetener, and they remained stable during the experiment. ***E***, Internal state ratings throughout the MRI scan showed that participants’ thirst significantly decreased, while hunger remained unchanged. Created with Biorender.com.

Participants completed an event-related probabilistic conditioning task, consisting of 144 trials (40 min duration). On each trial, participants were presented with a cue for 1.5 s, which included a pie chart indicating the probability of receiving either sweetener or sugar and images of beverages on the right and left side of the screen (Fig. 1*B*). The probabilities were 50–50% or 25–75%, and these were respected on all trials. The cue order and location of sweetener or sugar beverages was pseudorandomized across trials for each participant, resulting in six conditions (three probability levels, plus left- and right-side placement of the cue). Following the cue, participants were presented with the word “TASTE” and the image of a droplet for 3 s, which signaled the delivery of one of the liquids. Participants were instructed to hold the liquid in their mouth until seeing the word “SWALLOW”, which was presented for 5 s. During this time, participants used a sliding scale, ranging from 0 to 100 with anchors of “Sugar” and “Sweetener,” to indicate whether they had received sweetener or sugar (Fig. 1). Participants used a Current Designs 1-by-4 button box to move the computerized slider, where they used their index finger to move the slider left, their middle finger to move it right, and their ring finger to submit their response. Responses were used to derive both accuracy and confidence measures, where responses >51 were classified as correct and <51 incorrect. Confidence was calculated as the absolute difference of the response from the midpoint, which was divided by the range and multiplied by 100: [|response, 50|/50] × 100. The starting position of the slider was randomized across trials, which ensured that confidence ratings were decoupled from the distance the slider was moved. A fixation cross of variable duration (0.75, 1.12, 1.5, 1.9, or 2.25 s, pseudorandomized) was presented between each stimulus. After every 7–11 trials, a rinse was delivered to wash out the lemonade flavor.

In order to refill the pumps, two pauses, lasting ∼3–5 min, were added to the task. During these two pauses, a fixation cross was presented on the screen, so participants were not aware of the refilling.

### Neuroimaging—deterministic task

The second fMRI task was a deterministic conditioning paradigm that took 25 min to complete. During this task, participants were presented cues that indicated which liquid (sugar, sweetener, water) would be delivered (Fig. 1*C*). Water delivery was always respected, but on 50% of the sugar and sweetener trials, cues were covertly violated (i.e., if the cue indicated sweetener, sugar was delivered and vice versa). After each cue, the word “TASTE” was shown for 3 s, indicating the liquid delivery from the pump. Participants held the liquid in their mouth until the word “SWALLOW” appeared in addition to the question “How pleasant was the liquid?” (5 s duration). VAS pleasantness ratings were made using the button box. Confidence ratings were not collected because they may have caused participants to question the deterministic (100%) probabilities of the cues. As in the probabilistic task, each event was followed by a fixation cross of variable duration (0.75, 1.12, 1.5, 1.9, or 2.25 s, pseudorandomized).

Participants completed 75 trials, comprising five different conditions of 15 trials each: water delivery, sugar cue and delivery (sugar expected), sweetener cue and delivery (sweetener expected), sugar cue and sweetener delivery (sugar unexpected), and sweetener cue and sugar delivery (sweetener unexpected).

### Statistical analysis—behavior

Behavioral data were analyzed using linear mixed-effect modeling (LMMs) in R (v3.6.3), using the R package *nlme* (Pinheiro et al., 2023). To provide complementary information on satiety status during the fMRI scan, LMMs were estimated to determine the effects of time point and, when appropriate, flavor type on internal state and liking ratings. Reverse Helmert contrast coding was used to examine differences between time points, such that the mean of each level (i.e., time point) was compared with the mean of the previous levels.

For the probabilistic task, we examined the effect of flavor delivered (sugar, sweetener), probability level [unexpected (25%), chance (50%), expected (75%)], and their interaction on participant accuracy and confidence. Specifically, the probability level of a given trial was defined relative to the flavor delivered. That is, if a cue indicated 25% probability of sugar and 75% probability of sweetener and sugar was delivered, this would be considered unexpected. If the same cue was followed by delivery of the non-nutritive sweetener, this would be considered expected. For the probabilistic paradigm, we anticipated improved accuracy for high-probability flavors and poorer accuracy following low-probability ones, relative to chance. Probability levels were compared using nonorthogonal contrast coding, which compared both unexpected versus chance and expected versus chance trials. This model included random intercepts for flavor and probability level, which were nested within the participant's random effect. Results were corrected for multiple comparisons using a Bonferroni’s correction across the two models (*p* = 0.05/2 = 0.025).

For the deterministic task, we determined the effect of flavor delivered (sugar, sweetener), violation conditions (expected, unexpected), and their interaction on subjective pleasantness ratings. We predicted expectation would modulate pleasantness ratings during the deterministic paradigm, where the expectation of sugar would relate to greater pleasantness. Random intercepts for flavor type and violation were nested within the subject's random effect. Because expectancies relating to water trials were not manipulated in either task, they were excluded from behavioral analyses. All data met the assumptions of the aforementioned statistical tests.

### Statistical analysis—neuroimaging

Functional MRI data were preprocessed and analyzed using the SPM12 software (Wellcome Department of Cognitive Neurology) in MATLAB (MathWorks). Preprocessing included slice timing correction, within-subject image realignment to the mean volume, voxelwise weighted echo combination (summation based on local T_2_* measurements; Poser et al., 2006), coregistration of functional images with the T_1_-weighted anatomical scan, spatial normalization to the Montreal Neurological Institute (MNI) template, and spatial smoothing using a 6 mm full-width-at-half-maximum Gaussian kernel. The resulting time-series for each functional run were high-pass filtered (1/128 Hz), and serial autocorrelations were estimated using a FAST model. Following exclusions for quality control (e.g., excessive head motion, task nonresponsiveness), preprocessed data were available from *n* = 21 and *n* = 23 participants for the probabilistic and deterministic tasks, respectively.

For both tasks, images were analyzed in an event-related manner using a general linear model (GLM), and six nuisance regressors were included in the GLM to account for motion-related artifacts (rotation and translation in *X*, *Y*, *Z* planes). The probabilistic task included nine explanatory variables: the cue type (25% sugar and 75% sweetener, 50% sugar and 50% sweetener, 75% sugar and 25% sweetener) and the flavor delivered, depending on the probability of receiving it [unexpected (25%), uncertain (50%), and expected (75%) sugar delivery; unexpected (25%), uncertain (50%), and expected (75%) sweetener delivery]. Regressors of no interest were included for swallow events, water delivery, and refilling of the syringes. Contrasts were generated to assess the effects of cue and the flavor type on the BOLD response across differing probability levels. Specifically, we estimated contrasts for expected versus unexpected (e.g., 75% sugar vs 25% sugar), expected versus uncertain (e.g., 75% sugar vs 50% sugar), and unexpected versus uncertain (e.g., 25% sugar vs 50% sugar) sugar and sweetener cues, as well as their delivery. To examine potential differences in brain responses to each flavor, we computed three additional contrasts for (1) sweetener versus sugar delivery on uncertain trials (50–50% cues), (2) sugar delivery relative to the implicit baseline, and (3) sweetener delivery relative to the implicit baseline. Finally, we examined the parametric effect of confidence on BOLD responses to (1) sugar delivery, (2) sweetener delivery, and (3) sugar versus sweetener delivery. Confidence estimates were generated based on the participant's taste identification rating, regardless of the flavor delivered.

Nine explanatory variables were modeled for the deterministic task: (1) the cue (sweetener, sugar, water); (2) the flavor received across violation conditions (expected sugar, unexpected sugar, expected sweetener, unexpected sweetener, water); and (3) swallow events. Swallow events were considered a nuisance regressor. We included pleasantness ratings as a parametric modulator for sugar and sweetener delivery. To determine the effect of expectation on brain responses to each flavor, we generated four contrasts: expected versus unexpected sugar, expected versus unexpected sweetener, expected sugar versus expected sweetener, and unexpected sugar versus expected sweetener. To evaluate brain responses to the delivery of sugar and sweetener, regardless of expectation, we created two additional contrasts to compare flavor delivery to the implicit baseline.

**Figure 2.**
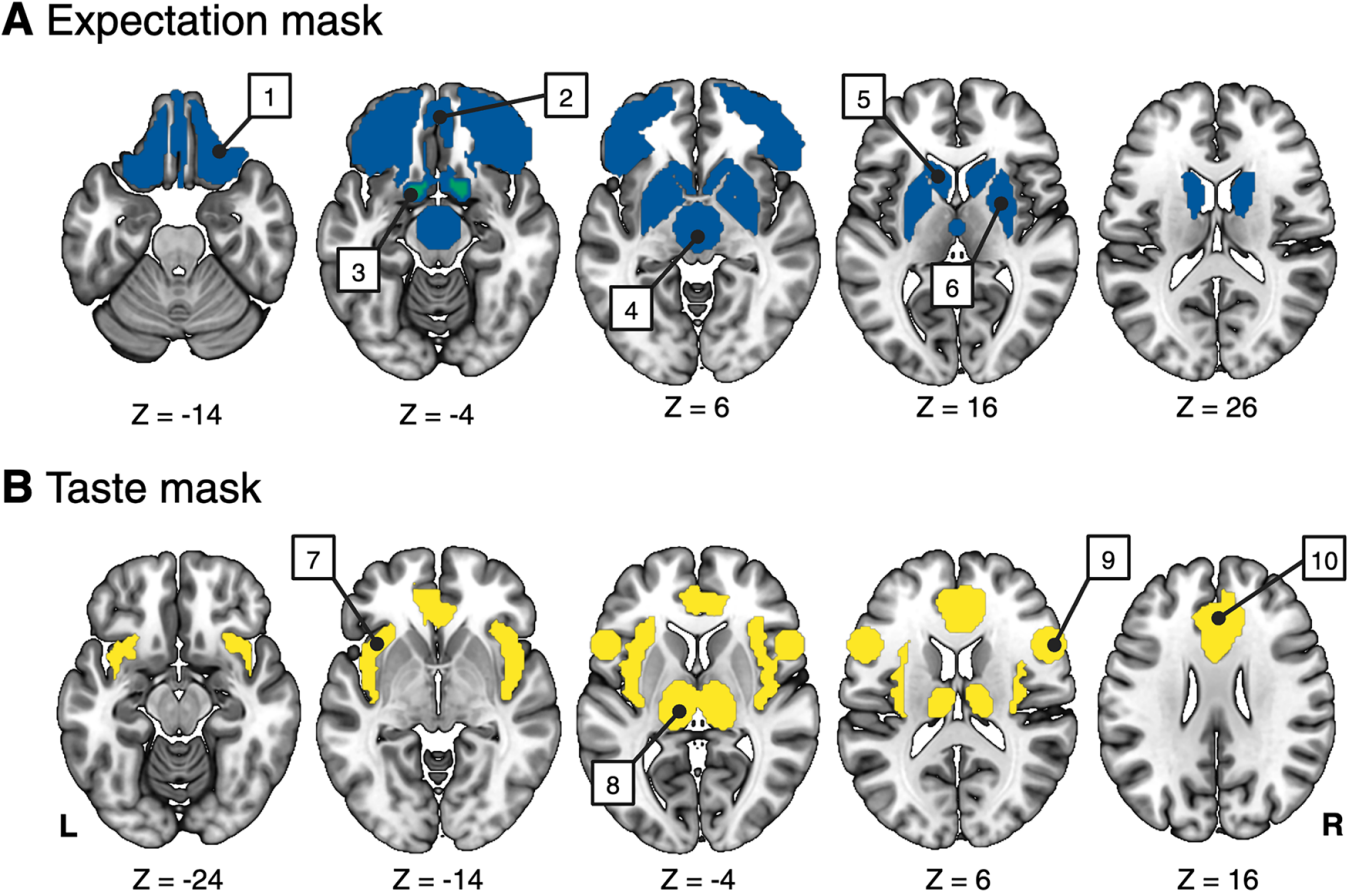
ROIs in taste and expectation masks. ***A***, The expectation mask was composed of bilateral basal ganglia and ventral frontal regions: 1, orbitofrontal cortex; 2, ventromedial prefrontal cortex; 3, nucleus accumbens; 4, midbrain; 5, caudate; 6, putamen. ***B***, The taste mask included bilateral regions that have been functionally implicated in flavor perception: 7, insula; 8, thalamus; 9, frontal operculum; and 10, anterior cingulate cortex. Created with Biorender.com.

Contrast estimates from the first-level contrasts were entered into a second-level effects analysis, using a one-sample *t* test (one-tailed) to compare activation levels in the condition of interest. Second-level analyses were initially conducted within a priori regions of interest (ROIs) before conducting an exploratory whole-brain analysis for each contrast of interest. ROIs were either generated or selected using the Wake Forest University Pick Atlas (Maldjian et al., 2003) and merged across two masks, one including regions implicated in expectation and the other taste processing (Fig. 2). The expectation mask was composed of bilateral basal ganglia and ventral frontal regions: orbitofrontal cortex, ventromedial prefrontal cortex, midbrain, putamen, caudate, and nucleus accumbens. The taste mask included bilateral regions that have been functionally implicated in flavor perception: insula, anterior cingulate cortex, thalamus, and frontal operculum. The nucleus accumbens ROI was inflated by 2; all other regions were generated without inflation. The midbrain and operculum regions were functionally defined based on prior literature, and all other regions were anatomically defined. For the midbrain, a 15 mm spherical region was generated from findings of Murray et al. (2008), with central coordinates of MNI*_X_*_,*Y*,*Z*_ = 0, −15, −9. The bilateral frontal operculum was estimated from the peak locations reported by Small et al. (2004), where an 11 mm sphere was centered on the following coordinates for the right and left hemisphere, respectively: MNI*_X_*_,*Y*,*Z*_ = 54, 15, 12 and MNI*_X_*_,*Y*,*Z*_ = −54, 15, 1.

A small-volume correction was applied to our ROI analyses, and statistical maps from the exploratory whole-brain analyses were adjusted for multiple tests using cluster-based correction (cluster-defining threshold of *p* < 0.001, FWE-corrected cluster *p* < 0.05, one-tailed). For additional discussion of statistical thresholding in SPM12 (Ashburner et al., 2015; Pauli et al., 2016), see Supplementary Material and Table S1, which reports ROI findings at a voxelwise threshold *p* < 0.0005 and FWE-corrected *p* < 0.025. This correction yields functional results that are statistically equivalent to a two-sided test (Chen et al., 2018).

## Results

### Demographics and internal state ratings

Demographic data from the final sample are reported in Table 1. The final sample was similar in age, BMI, and educational attainment to the participants (*n* = 71) who were ineligible based on their discrimination of sugar and sweetener. Liking ratings did not differ significantly between flavors (*p* = 0.80), and they did not change from baseline (*p* = 0.41; Fig. 1*D*). A flavor-by-time interaction effect was nonsignificant (model *χ*^2^_(10)_ = 2.74; *p* = 0.25), indicating that liking ratings for each flavor remained similar for the duration of the experiment (Fig. 1*E*). Thirst ratings decreased across time points [mid-MRI 2 vs pre-MRI: *B*(SE) = −32.65(7.15); *p* < 0.0001; post-MRI vs pre-MRI and mid-MRI, *B*(SE) = −18.49(6.20); *p* = 0.0047], whereas hunger levels remained stable (all *p*’s > 0.45).

**Table 1.** Demographic information

Characteristic	Ineligible screened sample		fMRI sample	
Sex (F/M)	35/36		19/8	
	M	SD	M	SD
Age (years)	23.55	3.96	24.25	2.94
BMI (kg/m^2^)	21.71	1.83	21.34	1.67
Years of education (years)	15.51	1.95	15.75	1.33
Patient Health Questionnaire-9	-	-	2.33	2.65
Three-Factor Eating Questionnaire-Revised 18				
Cognitive restraint	-	-	13.79	3.72
Uncontrolled eating	-	-	17.62	4.11
Emotional eating	-	-	6.17	2.37
Kessler Psychological Distress Scale	-	-	13.46	3.66
Recent Physical Activity Questionnaire	-	-		
METhrS	-	-	16.8	6.95
Time	-	-	20.3	8.86

MeThrs, estimated daily total energy expenditure, which is the product of the total reported duration of activity (hours) and intensity (sedentary, light, moderate, vigorous). Time, total time spent in any activity or sleep (hours/day).

### Behavioral results

**Figure 3.**
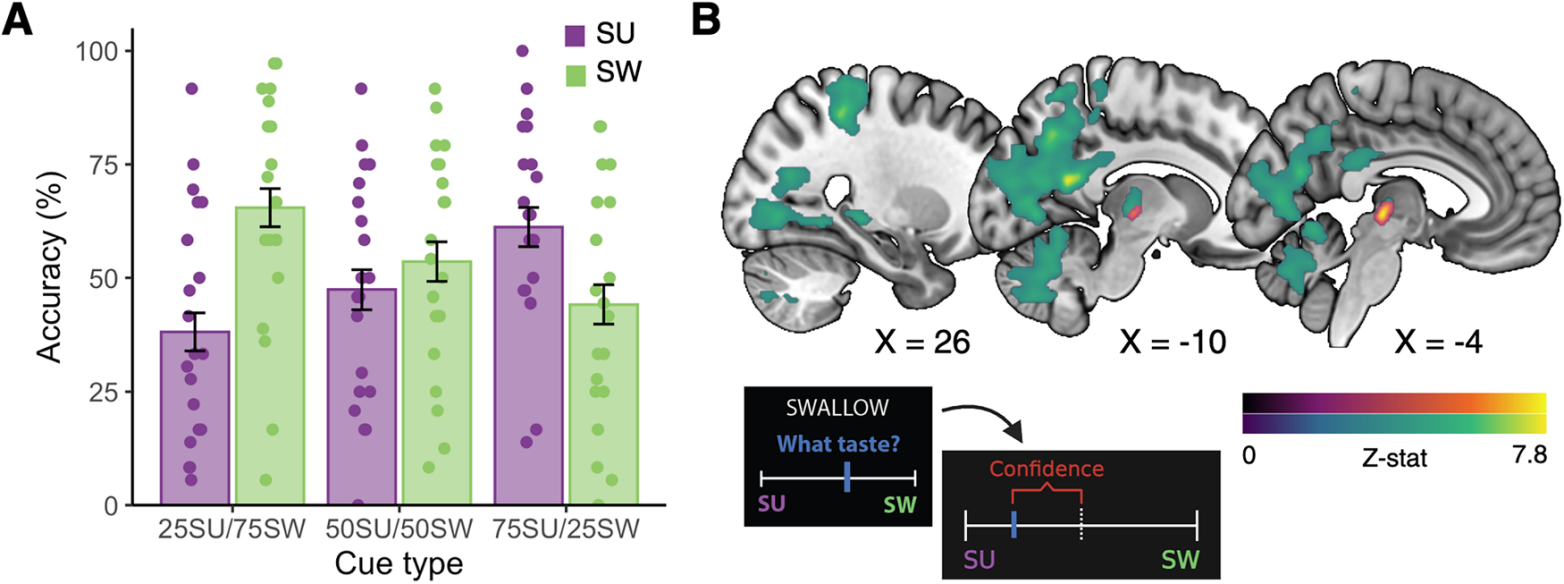
Probabilistic task performance and neural encoding of confidence. ***A***, The probability of flavor delivery significantly affected accuracy, which was greater for expected flavors and nominally lower for unexpected flavors, relative to chance. Visually, this is evidenced by the ascending and descending accuracy values for sugar and sweetener, respectively, across cue types. Accuracy did not differ across flavor types. Colors depict the flavor delivered. Error bars, 95% confidence interval. ***B***, The parametric effect of trial-by-trial confidence differed between sugar and non-nutritive sweetener trials in both our a priori ROI analyses (purple/orange color bar; voxelwise *p* < 0.001; small volume corrected *p* < 0.05) and whole-brain analyses (blue/green color bar; voxelwise *p* < 0.001; FEW-corrected cluster probability *p* < 0.05). For details on cluster size, coordinates, and associated test statistics of these one-tailed *t* tests, see Table 2 and Table S1. Confidence was calculated from taste identification ratings as the absolute difference of the response (blue) from the midpoint (dashed line), which was divided by the range and scaled by 100.

LMMs for the probabilistic task examined the effect of task conditions on flavor identification accuracy and confidence, whereas deterministic task models examined these effects on pleasantness (see Materials and Methods). On the probabilistic task, the main effect of flavor type on accuracy was nonsignificant, indicating that participants were unable to consistently distinguish between the flavors. Moreover, a significant main effect of the probability level indicated that participants were nominally less accurate for unexpected trials relative to chance [*B*(SE) = −5.74(2.58); *p* = 0.032], and they were more accurate on trials when the expected flavor was delivered as compared with chance [*B*(SE) = 9.22(2.59); *p* = 0.001; Fig. 3*A*]. This would suggest that the task successfully manipulated participants’ expectations regarding flavor delivery. All interaction effects on accuracy were nonsignificant. The main and interaction effects of the flavor type and probability level were not significantly related to confidence ratings (all *p*’s > 0.14).

**Figure 4.**
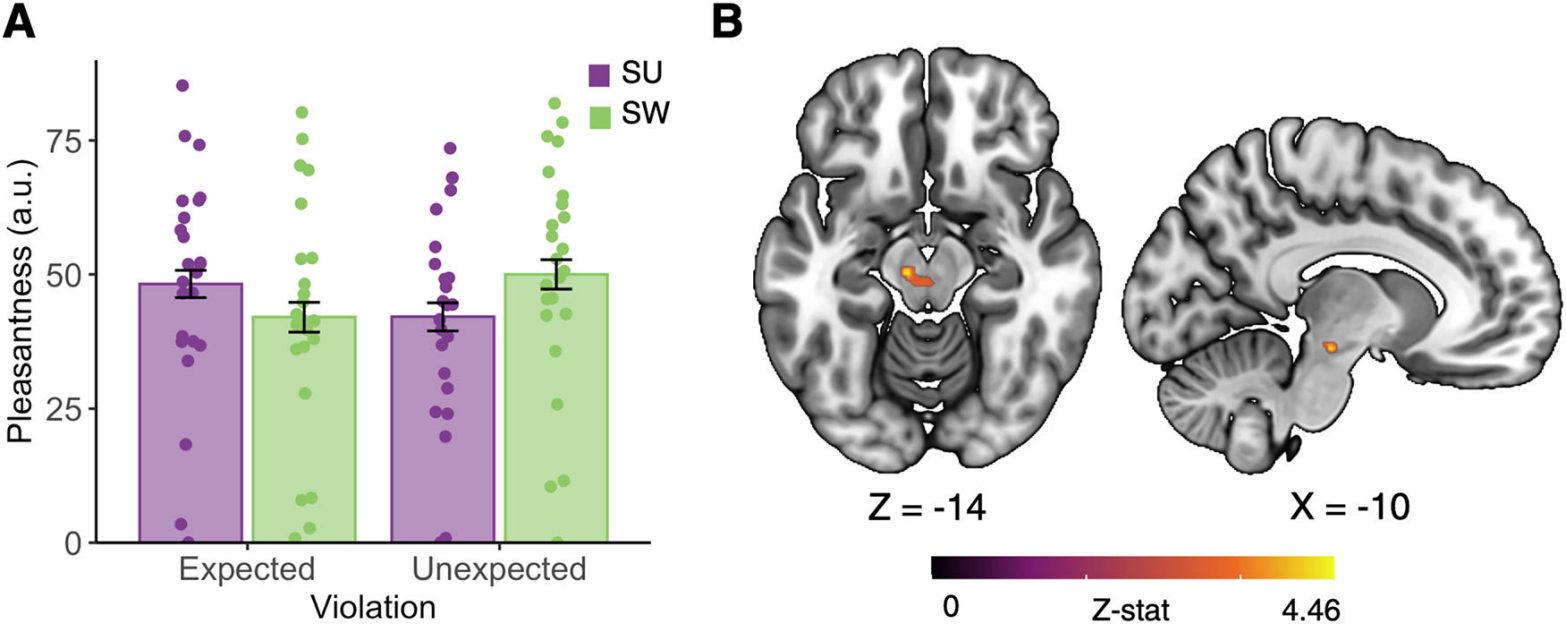
Expectation of sugar augments pleasantness ratings and midbrain responses to non-nutritive sweetener. ***A***, A flavor-by-violation interaction indicated that participants found unexpected non-nutritive sweetener to be more pleasant than unexpected sugar. That is, participants rated non-nutritive sweetener as more pleasant when they thought they had received sugar relative to when they thought they received sweetener (and vice versa for sugar). Colors depict the flavor delivered. Error bars, 95% confidence interval. ***B***, This effect corresponded with increased left midbrain activation for unexpected versus expected non-nutritive sweetener delivery (*t*-stat = 5.78; peak = 0.024, 46 voxels). Created with Biorender.com.

LMMs for the deterministic task showed that the main effects of flavor and violation of expectation on pleasantness ratings were nonsignificant (*p*’s > 0.05); however, a significant flavor-by-violation (of expectation) interaction effect indicated that pleasantness of the non-nutritive versus sugar-sweetened lemonade was significantly greater on unexpected versus expected trials [*B*(SE) = 14.06(1.74); *p* < 0.001]. That is, participants found non-nutritive sweetener to be more pleasant when they were expecting sugar than when they were expecting sweetener, and the opposite was observed for sugar (Fig. 4*A*). This can be conceptualized as a significant difference of differences in pleasantness ratings across unexpected and expected delivery of each flavor. Inclusion of the interaction term significantly improved model fit (*χ*^2^_(1)_ = 64.56; *p* < 0.001).

### Neuroimaging results—probabilistic task

During the probabilistic conditioning task, participants’ expectations were manipulated by presenting visual cues depicting varying probability levels (25, 50, 75%) of either sugar- or artificially sweetened beverage delivery. We were therefore able to examine brain responses to high-probability versus uncertain sugar (75% sugar cue vs 50% sugar cue) and non-nutritive sweetener (75% sweetener cue vs 50% sweetener cue), as well as low-probability versus uncertain sugar (25% sugar cue vs 50% sugar cue) and sweetener (25% sweetener cue vs 50% sweetener cue; Fig. 1*A*,*B*). However, it should be noted that the high-probability cues for one flavor also conveyed a low probability of the other. Our primary ROI analyses indicated that brain responses across striatal, medial frontal, orbitofrontal, and midbrain regions were not significantly different across probability levels for both sugar and non-nutritive sweetener. The main effect of the probability level was largely nonsignificant at the whole-brain level; however, left superior occipital, lingual gyrus, and cerebellum activation was greater for uncertain (50% probability) cues as compared with the other probability levels (Table S2).

When examining neural responses to flavor delivery, sugar-sweetened beverages elicited increased left putamen activation, whereas artificially sweetened beverages increased brain responses in several regions that have established roles in gustatory and reward processing (Table 2, Fig. S1). The main effect of the flavor type across all probability levels was not significant, suggesting markedly similar brain responses to sugar and non-nutritive sweetener delivery, which mirrored participants’ behavioral ratings of these flavors. However, exploratory whole-brain analyses indicated that, on maximally uncertain trials (50%), left superior parietal activation was increased following non-nutritive sweetener versus sugar delivery (Table S2).

**Table 2.** ROI brain responses during the probabilistic conditioning task

Contrast	Mask	Side	Region	Peak MNI coordinates			Size (voxels)	*Z* statistic	Peak pFWE
				*X*	*Y*	*Z*			
SU taste > baseline	Expect	L	Putamen	−26	−4	−2	151	4.20	0.041
SW taste > baseline	Taste	R	Thalamus (extends bilaterally)	2	−20	4	696	5.06	0.001
		R	Precentral gyrus/IFG pars opercularis	56	8	18	188	4.68	0.005
		R	Anterior insula	36	−2	14	422	4.58	0.007
		L	Anterior insula	−38	−16	6	678	4.51	0.01
	Expect	R	Thalamus	2	−20	4	886	5.06	0.001
		R	Putamen/pallidum	30	−10	−6	705	4.57	0.011
		L	Putamen	−26	−2	−6	476	4.53	0.013
Average confidence	Taste	R	Posterior insula	42	0	−12	31	4.04	0.037
		L	Thalamus	−6	−30	8	5	4.04	0.037
		R	Thalamus	6	−30	6	17	4.01	0.042
SU taste confidence	Taste	L	Bilateral thalamus	−12	−20	8	1,066	4.55	0.006
		R	Posterior insula	36	−6	14	85	4.52	0.007
		L	Posterior insula	−36	−10	14	182	4.38	0.012
		L	Pars opercularis	−58	10	4	36	4.21	0.021
		L	Parietal operculum	−32	−32	20	9	4.06	0.036
SU taste > SW taste confidence	Taste	L	Left thalamus	−4	−22	0	172	4.97	0.001
		R	Right thalamus	6	−20	0	131	4.48	0.006

MNI coordinates represent the peak voxel within each cluster. Brain areas comprising expectation and taste masks are illustrated in Figure 2. Clusters within these ROIs were identified using a small-volume correction (voxelwise *p* < 0.001), and peak *p* < 0.05 were considered statistically significant. Peak *Z* statistics are reported. SU, sugar-sweetened beverage; SW, non-nutritive sweetened beverage.

The parametric effect of trial-wise confidence across all trial types was associated with greater activation in primary sensory areas, including the bilateral thalamus and right posterior insula (ROI analyses; Table 2). Moreover, as confidence increased, bilateral thalamic responses following sugar versus non-nutritive sweetener delivery also increased; however, there were no significant differences across probability levels. At the whole-brain level, increasing confidence was related to increased activation across three large clusters, spanning hippocampal, parahippocampal, primary sensory, and gustatory regions (Table S2, Fig. S3). Moreover, the parametric effect of confidence was related to significantly increased activation in one large cluster, spanning superior parietal, inferior parietal, and thalamic regions, following sugar delivery relative to non-nutritive sweetener (Fig. 3*B*; Table S2). Perceptual decision-making experiments have implicated hippocampal and parietal regions in the estimation of choice uncertainty (Kiani and Shadlen, 2009; Rutishauser et al., 2015), and our findings suggest that this circuit may also code confidence estimates in relation to gustatory stimuli.

### Neuroimaging results—deterministic task

Following the probabilistic task, participants performed a deterministic conditioning paradigm in which they viewed cues for either sugar, non-nutritive sweetener or water beverages, and these were followed by a 0.9 ml delivery of the cued liquid. However, the cues were covertly violated on 50% of sugar and sweetener trials (i.e., a sugar cue was succeeded by sweetener delivery and vice versa; see Materials and Methods). As in the probabilistic task, delivery of both sugar- and artificially sweetened liquids was related to increased activation of gustatory and primary sensory regions relative to the implicit baseline (Table 3, Fig. S2). Although brain responses did not differ across unexpected versus expected sugar trials, we observed increased midbrain activation on unexpected versus expected sweetener trials in our ROI analysis (Fig. 4*B*; MNI*_X_*_,*Y*,*Z*_ = −10, −22, −14; *Z* = 4.46; *p* = 0.024; small-volume corrected). This may suggest that the dopaminergic midbrain preferentially encodes the value of sweet flavor when nutritive value is expected.

**Table 3.** ROI brain responses during the deterministic conditioning task

Contrast	Mask	Side	Region	Peak MNI coordinates			Size (voxels)	*Z* statistic	Peak pFWE
				*X*	*Y*	*Z*			
Unexpected SW > expected SW taste	Expect	L	Midbrain, SN pars compacta	−10	−22	−14	46	4.46	0.024
SU taste > baseline	Expect	L	Pallidum, putamen	−26	−14	−4	89	4.25	0.022
	Taste	L	Posterior insula	−36	−12	14	184	4.31	0.012
		R	Posterior insula	36	−6	−14	125	4.27	0.015
		R	Thalamus	12	−16	6	186	3.98	0.041
		L	Thalamus	−10	−20	0	152	3.93	0.048
SW taste > baseline	Taste	L	Posterior insula	−36	−12	14	139	4.18	0.021
		R	Posterior insula	36	−6	12	91	4.12	0.025
Pleasantness SU taste	Taste	L	Posterior insula, central operculum	−38	−12	18	164	4.70	0.004
		R	Thalamus	12	−14	6	175	4.21	0.024
		R	Posterior insula, central operculum	40	−8	22	88	4.11	0.035
		L	Parietal operculum, posterior insula	−32	−32	20	23	4.09	0.037

MNI coordinates represent the peak voxel within each cluster. Brain areas comprising expectation and taste masks are illustrated in Figure 2. Clusters within these ROIs were identified using a small-volume correction (voxelwise *p* < 0.001); peak *p* < 0.05 were considered statistically significant. Peak *Z* statistics are reported. SU, sugar-sweetened beverage; SW, non-nutritive sweetened beverage.

Since our behavioral results suggested that expectation modulates the subjective pleasantness of sweet flavor, we interrogated the parametric effect of pleasantness on brain responses following liquid delivery. As pleasantness increased, brain responses to sugar also increased in the bilateral insula and right thalamus, and this effect did not differ across expected versus unexpected trials (ROI analyses; Table 3). Exploratory whole-brain analyses indicated that these effects extended to two large clusters, which encompassed several regions known to be associated with sweet flavor perception and interoception (e.g., postcentral gyrus, basal ganglia, brainstem nuclei, cerebellum; Table S3, Fig. S4). Although pleasantness ratings did not alter brain responses to non-nutritive sweetener, the parametric effect of pleasantness differentially modulated lateral occipital responses to sugar versus non-nutritive sweetener (Table S3). This effect did not differ by expectation.

## Discussion

The present study implemented two conditioning paradigms to characterize how expectations of sugar or non-nutritive sweetener affect the neural coding of sweet flavor. Critically, to control for differences in the sensory experience of each flavor, we assessed these processes in healthy adults who were unable to reliably distinguish sugar- and non-nutritive sweetened beverages. Analysis of both behavioral and neuroimaging data yielded three key results. First, in a probabilistic conditioning task, the probability level significantly affected flavor identification accuracy, and participant's confidence in their choice was related to increased brain responses across primary sensory, hippocampal, and parietal clusters. Second, during the deterministic task, expectation had opposing effects on the pleasantness of non-nutritive sweetener and sugar, where the expectation of sugar was associated with enhanced subjective pleasantness when participants unknowingly received non-nutritive sweetener. Increased pleasantness ratings scaled with brain responses to sugar, but not sweetener, across a putative gustatory network. Finally, the expectation of sugar significantly augmented midbrain responses to covert sweetener delivery, suggesting that the expectation of nutritive value accounts, at least in part, for the distinct reinforcing effects of caloric and noncaloric sweeteners.

Findings from our probabilistic conditioning paradigm indicated that participants relied primarily on predictive cues to distinguish sugar and non-nutritive sweetener. When examining the main effects of flavor and probability level on accuracy, we found that only the predictive information significantly altered accuracy, where participants had improved performance for expected flavors and nominally poorer accuracy for unexpected ones. However, these cues were not associated with either differences in mesolimbic activation or with participants’ confidence in their choices. The latter would suggest that participants overweighted gustatory information relative to imprecise predictive information when generating their confidence estimates. Indeed, the parametric effect of confidence was related to increased thalamic and insula activation following flavor delivery in our ROI analyses, as well as greater bilateral (para)hippocampal activation in our exploratory whole-brain analysis. Although meta-analyses support the role of the parahippocampal gyrus in retrospective confidence judgments (Vaccaro and Fleming, 2018; Martín-Luengo et al., 2021), the present findings, to our knowledge, are the first to implicate this region in confidence estimation during gustatory decision-making. We also identified clusters in the posterior thalamus, superior parietal cortex, and occipitoparietal junction that preferentially tracked confidence following sugar versus non-nutritive sweetener delivery. Several lines of evidence suggest that the parietal cortex simultaneously accumulates evidence for and codes confidence in perceptual decisions (Kiani and Shadlen, 2009; Gherman and Philiastides, 2015), particularly under conditions of uncertainty. Our findings broadly align with this interpretation, yet it remains unclear as to why such an effect would be specific to sugar delivery.

The deterministic conditioning paradigm further demonstrated that predictive information modulates the pleasantness of sweet flavor, suggesting that the expected value of sugar and sweetener can override their sensory properties. The direction of this effect differed between sugar and non-nutritive sweetener, where participants rated unexpected sweetener as more pleasant than expected sweetener and vice versa for sugar. That is, when sweetener delivery was preceded by sugar cues, participants rated the flavor as more palatable than when they expected sweetener. Similarly, participants found sweetener-cued sugar delivery to be less palatable than expected sugar. Moreover, in our exploratory analyses, the parametric effect of pleasantness was associated with greater activation across a putative gustatory network (e.g., thalamus, posterior insula, postcentral gyrus, and basal ganglia) following sugar—but not sweetener—delivery. Several lines of evidence support a conserved preference for caloric versus noncaloric sweetener across species (Sclafani, 2004; Yeomans et al., 2008), which likely results from postingestive signals (e.g., via hepatic portal vein glucose sensing, glucose metabolism) that reinforce its nutritive value in the brain (Oliveira-Maia et al., 2011; Zhang et al., 2018; de Araujo et al., 2020). Existing flavor-nutrient conditioning in our sample would augment the expected value of sugar relative to sweetener, and indeed, our results suggest that expectations surrounding the nutritive value of a sweet flavor significantly alter its palatability.

Intriguingly, we observed greater midbrain activation for unexpected versus expected sweetener delivery, yet there were no differences for unexpected sugar delivery. This may be understood within a predictive processing framework, in which unreliable sensory information has tipped the balance in favor of more precise predictive information, and consequently, the expectation of nutritive value from a sweet flavor increases its subjective value. Preclinical models indicate that the reinforcing effects of sugar are largely mediated by dorsal striatal circuitry, and peak activation of our midbrain cluster fell within the left substantia nigra (SN), which projects to the dorsolateral striatum via the nigrostriatal pathway (Haber, 2011). Activation of dopaminergic D1 neurons within the dorsal striatum–SN pathway has been shown to elicit calorie-seeking in rodents, even when sugar is paired with an aversive flavor (Tellez et al., 2016). These effects may relate to the broader functions of the SN, which include both sensing gut-induced reward from right-lateralized vagal afferents (Han et al., 2018) and estimation of action-based value to support goal-directed behavior (García-García et al., 2017). Although we cannot exclude the possibility that our cluster spanned additional midbrain nuclei, our findings might suggest that the SN encodes the expectation of sugar-associated calories, which may, in turn, facilitate action selection to obtain a calorific reward.

Despite having notable strengths, several limitations of this study should be considered. First, we recruited a modest sample size of individuals who could not reliably distinguish between sugar- and non-nutritive–sweetened beverages, and it remains unclear if our findings will generalize to the broader population. Encouragingly, previous studies have found that individuals poorly discriminate between similar flavors (Hettinger et al., 1999), with accuracy rates of only 66% when identifying an artificially sweetened beverage (Delogu et al., 2016). This may suggest that our sample reflects a substantial proportion of the general population, but replication efforts in larger samples and preregistered protocols are warranted. Second, our probabilistic task used cue stimuli that presented reciprocal probabilities for each flavor (i.e., the high-probability cue for sugar conveyed the same information as the low-probability cue for sweetener). As such, neural responses to expected and unexpected flavors could not be fully dissociated from one another. The use of these cues increased the efficiency of our event-related paradigm, yet future studies may wish to include cues that depict probabilities for only one flavor at a time. Third, participants were provided water rinses every 7–11 trials, whereas trial-wise rinses would have further minimized sensory adaption and contamination across trials. Future efforts that implement trial-by-trial rinses will be useful for determining the generalizability of our findings. Finally, our sample was restricted to lean volunteers, which limited our ability to examine neural responses to sweet flavor across the full BMI range. Alterations in putative sensory and reward circuitry have been identified in individuals with obesity (Carnell et al., 2012; Medic et al., 2016; Avery et al., 2017; Makaronidis and Batterham, 2018), and our findings may guide future efforts to delineate the specific role of reward expectation in those struggling with elevated BMI and overeating.

Taken together, our findings advance understanding of the neurocognitive mechanisms that underlie the rewarding effects of sweet flavor, which are largely driven by expectation when sensory information is unreliable. This extends prior evidence of expectancy modulating subjective ratings of and neural responses to sweet (Woods et al., 2011; Wilton et al., 2019) and bitter taste (Nitschke et al., 2006). The reinforcing effects of sugar appeared to trump those of non-nutritive sweetener, and this likely reflects flavor-nutrient conditioning and the evolutionarily conserved drive for calories. These results underscore the role of expectancies in guiding food choice behavior, which may open new avenues for future dietary interventions. Such efforts may wish to emphasize the selection of nutrient-rich, as opposed to calorie-free, food options with minimal added sugars, which can promote sustained dietary change (U.S. Department of Agriculture and U.S. Department of Health and Human Services, 2020).
